# Melanin-Associated Synthesis of SERS-Active Nanostructures and the Application for Monitoring of Intracellular Melanogenesis

**DOI:** 10.3390/nano7030070

**Published:** 2017-03-20

**Authors:** Haixin Dong, Zhiming Liu, Huiqing Zhong, Hui Yang, Yan Zhou, Yuqing Hou, Jia Long, Jin Lin, Zhouyi Guo

**Affiliations:** 1 SATCM Third Grade Laboratory of Chinese Medicine and Photonics Technology, College of Biophotonics, South China Normal University, Guangzhou 510631, China; dhx98@sina.com (H.D.);honghq@scnu.edu.cn (H.Z.); 15322072000@163.com (Y.H.); 2 Infinitus (China) Company Ltd., Guangzhou 510665, China; Youky.Yang@infinitus-int.com (H.Y.); dhx98@tom.com (Y.Z.); Jia.Long@infinitus-int.com (J.L.); Jin.Lin@infinitus-int.com (J.L.)

**Keywords:** surface-enhanced Raman scattering, melanin–Ag nanocomposites, bioimaging, melanogenesis

## Abstract

Melanin plays an indispensable role in the human body. It serves as a biological reducer for the green synthesis of precious metal nanoparticles. Melanin–Ag nanocomposites were successfully produced which exhibited very strong surface-enhanced Raman scattering (SERS) effect because of the reducibility property of melanin. A melanin–Ag composite structure was synthesized in situ in melanin cells, and SERS technique was performed for the rapid imaging and quantitative assay of intracellular melanin. This imaging technique was also used to successfully trace the formation and secretion of intracellular melanin after stimulation with melanin-stimulating hormones. Based on the self-reducing property of melanin, the proposed SERS imaging method can provide potentially powerful analytical detection tools to study the biological functions of melanin and to prevent and cure melanin-related diseases.

## 1. Introduction

Melanin is a ubiquitous pigment in the biological system, and is one of the most important chromophores in the human body. Its presence is associated with human skin, hair, and eye color [1]. The major biological functions of melanin include photo-protection, photoreceptor shielding, thermoregulation, camouflage, and display [2,3]. The synthesis and secretion of intracellular melanin can be more accurately revealed by proper melanin detection and real-time imaging of intracellular melanin. Among conventional methods, optical detection techniques such as absorption spectroscopy, fluorescence spectrometry, and Raman scattering technique can rapidly and sensitively detect melanin, and quantitative and qualitative analyses of melanin can be achieved using spectral data [4,5,6]. Melanin has strong absorption within the ultraviolet-visible (UV-Vis) range, and its absorption decreases with increasing wavelength. However, the absorption spectra of melanin have low specificity; thus, no significant differences were observed compared with the spectral characteristics of other chromophores (e.g., hematoporphyrin) in the skin. Similarly, melanin stimulated by near-infrared light can emit fluorescence, but the spectrum is very wide and is prone to overlap with other biological components. By contrast, Raman scattering can provide molecular fingerprint-type signatures about the molecular composition through a narrow bandwidth spectrum [7]. However, normal Raman scattering signals are so weak that obtaining identifiable melanin Raman signals is generally time-consuming. Although Raman spectra of different types of melanins have been recorded before [6], so far the use of Raman scattering technique for the intracellular imaging of melanin has not been reported.

Recently, metal nanostructures synthesized by green methods have attracted wide attention [8,9]. Materials derived from biological systems such as plant extracts, microorganisms, and eukaryotic cells have proven to be effective and feasible bioreductive agents for the green synthesis of Ag nanoparticles (AgNPs), gold nanoparticles, and iron nanoparticles [10,11,12,13,14,15,16,17,18,19]. Melanogenesis begins with l-dihydroxyphenylalanine (l-DOPA) acquired from the hydroxylation of l-tyrosine, and is followed by a series of redox polymerization reactions [20]. This structure, which is rich in phenolic hydroxyl groups, gives melanin its strong reducibility. Melanin has recently been used as a novel reducer from a biological source for the green synthesis of precious metal nanoparticles. l-DOPA can enhance the intracellular synthesis efficiency of melanin by using a common biotech *Yarrowia lipolytica* (NCIM 3590 and 3589) with poorly expressed melanin, and researchers successfully synthesized spherical Au/Ag nanoparticles [21,22] In the reduction process, phenolic hydroxyl groups of melanin were transformed into quinonyl groups. Consequently, precious metal salts were transformed into elemental metals. In another study, they found *Yarrowia lipolytica* (NCYC 789), a marine yeast, and they completed the biosynthesis of AgNPs which exhibited high antibacterial activity [23]. In addition to yeast, Kiran et al. [24] found that *Nocardiopsis alba* MSA10—a marine actinomycete—secreted a large amount of melanin. By using melanin separated from the culture medium, they successfully completed the rapid green synthesis of AgNPs. The produced AgNPs were capable of linking to the amino groups of melanin, thereby facilitating the formation of stable nanocomposites [24]. Raman spectroscopy revealed that the Raman signals of the molecular structure of melanin could be significantly enhanced after deposition onto the metal nanoparticles; i.e., surface-enhanced Raman scattering (SERS) effect [25]. Thus, SERS technique can be effectively used for the rapid detection of melanin in the skin.

In the present study, the SERS technique is used to rapidly detect intracellular melanin and to monitor the synthesis and secretion of melanin in real-time. First, industrially synthesized melanin and silver–ammonia complex were used as precursors. Test-tube experiment confirmed that melanin–Ag nanocomposites were generated. Then, considering the reducibility of melanin, melanin–Ag nanocomposites were synthesized in situ in melanocytes, and the strongly enhanced melanin Raman signals were used to localize intracellular melanin by SERS imaging for the first time. Last, we monitored the synthesis and secretion of intracellular melanin by SERS in real-time.

## 2. Materials and Methods

### 2.1. Materials

Murine B16-F10 melanoma cells were obtained from Laboratory Animal Center of Sun Yat-sen University, (Guangzhou, China). RPMI-1640 medium and fetal bovine serum (FBS) were purchased from GIBCO (Grand Island, NY, USA). Melanin (alcohol soluble), 3-isobutyl-1-methylxanthine (IBMX), silver nitrate (AgNO_3_), and chloroauric acid (HAuC1_4_) were obtained from Aladdin (Shanghai, China). Other reagents were of analytical grade and were used as received without further purification. Deionized water (Milli-Q System, Millipore, Billerica, MA, USA) was used in all experiments.

### 2.2. Characterization

The UV-Vis spectra of the nanostructures were characterized using an UV-Vis spectrometer (UV-6300, MAPADA, Shanghai, China) with a1 cm quartz cuvette. Transmission electron microscopy (TEM) analysis was performed using a JEM-2010HR transmission electron microscope (JEOL, Tokyo, Japan) operated at 120 kV equipped with an energy-dispersive X-ray (EDX) analyzer. Raman spectra were collected using a Renishaw in via microspectrometer (In via, Renishaw Co., Derbyshire, UK) with excitation wavelengths of 514.5 and 785 nm.

### 2.3. Test-Tube Experiments

Preparation of silver–ammonia complex: first, 10 mL silver nitrate solution (50 g/L) was poured into a beaker. Aqueous ammonia was added into the beaker drop wise while shaking until the precipitated particles disappeared. Second, 10 mL silver nitrate solution (50 g/L) was added drop wise into the beaker until the liquid became very pale (milky color). The final production was filtered by 0.22 µm filter membranes.

Preparation of melanin–Ag nanocomposites: melanin was dissolved in ethylene glycol, and a 20 μg/mL solution was prepared. Melanin solution (4 mL) was poured into a beaker and heated in a water-bath at 56 °C. Silver ammonia solution was added slowly, and the reaction was stopped after 5 min. The action product was centrifuged for 10 min at 6000 rpm three times. The precipitate was re-suspended with ethylene glycol.

### 2.4. In Vitro Experiments

In situ synthesis and SERS imaging of intracellular nanocomposites: B16-F10 cells were kept in RPMI-1640 culture medium (containing 10% FBS) and placed in a CO_2_ incubator (at 37 *°*C, containing 5% CO_2_) for adherent culture. The cells grew until 70%–80% merged. The cells were dissociated with pancreatin and inoculated in a sterile coverslip. After the cells reached a certain density, the coverslip was removed, rinsed three times with phosphate buffered saline (PBS), fixed for 5 min with 4% neutral formalin, rinsed with ultrapure water, soaked in silver ammonia solution, placed in a wet box at 56 °C, hatched for 15 min, rinsed with ultrapure water, hatched for 4 min after addition of 5 mM chloroauric acid, treated for 2 min with 5% sodium thiosulfate solution, and rinsed with ultrapure water. Then, the treated cells were placed under the Raman microscope and stimulated with 785 nm laser (power: ~10 mW). Streamline scanning mode was selected, with a central wavenumber of 1350 cm^−1^. The Raman range of 1000–1700 cm^−1^ wasselected for imaging.

SERS-based analysis of the generation and secretion of intracellular melanin: after B16-F10 cells were attached, the samples were treated with melanin-stimulating hormone (IBMX). At different time points (0, 6, 12, and 24 h), intracellular nanocomposites were structured in situ and quickly detected by SERS imaging (under streamline scanning mode with 2 s exposure).

## 3. Results and Discussion

### 3.1. Synthesis and Characterization of Melanin–Ag Nanocomposites

**Figure 1:**
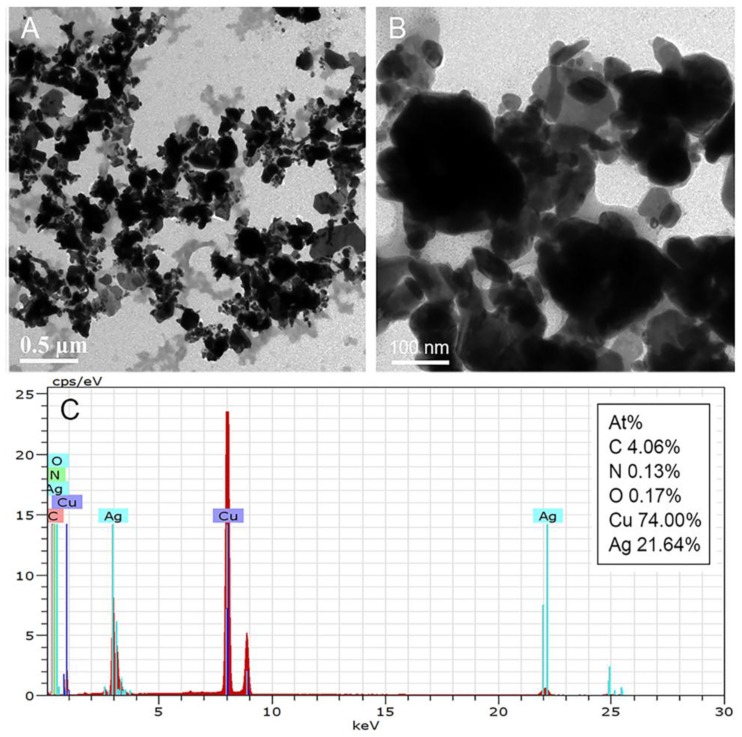
Characterization of the nanostructures. (**A**,**B**) Transmission electron microscopy (TEM) images of melanin–Ag nanocomposites at different magnifications. (**C**) Energy-dispersive X-ray spectroscopy (EDX) pattern of the nanostructures.

To verify the reducibility of melanin, a test-tube experiment was performed with industrially synthesized melanin and silver–ammonia complex as precursors. Figure 1A,B shows the morphological characterization of the melanin–Ag nanocomposites. Deep black silver nanoparticles were of different sizes (200 nm on average), and a number of protrusions outside of the deep black particles were observed. In TEM images with higher magnification, the Ag-nanostructure was built by various smaller silver nanoparticles. Non-stacked silver particles were deposited, thereby forming protrusions on the surface. The light gray substance outside of the Ag nanostructure is composed of attached melanin molecules. EDX spectroscopy revealed that aside from the widely existing Ag, three elements (namely, C, O, and N) are also present, further proving the successful synthesis of melanin–Ag nanocomposites.

**Figure 2:**
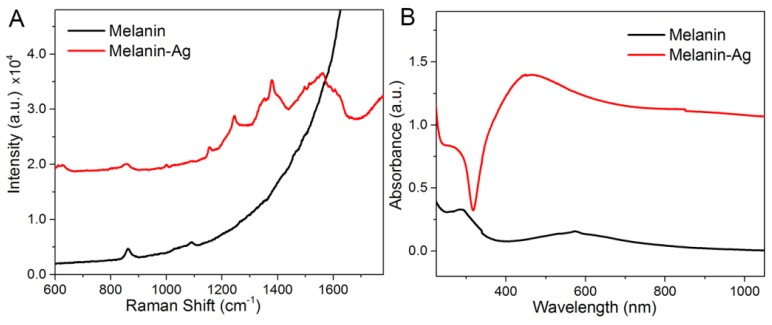
(**A**) Raman spectra under 514.5 nm excitation and (**B**) the UV-Vis absorbance spectra of the nanomaterials.

**Figure 3:**
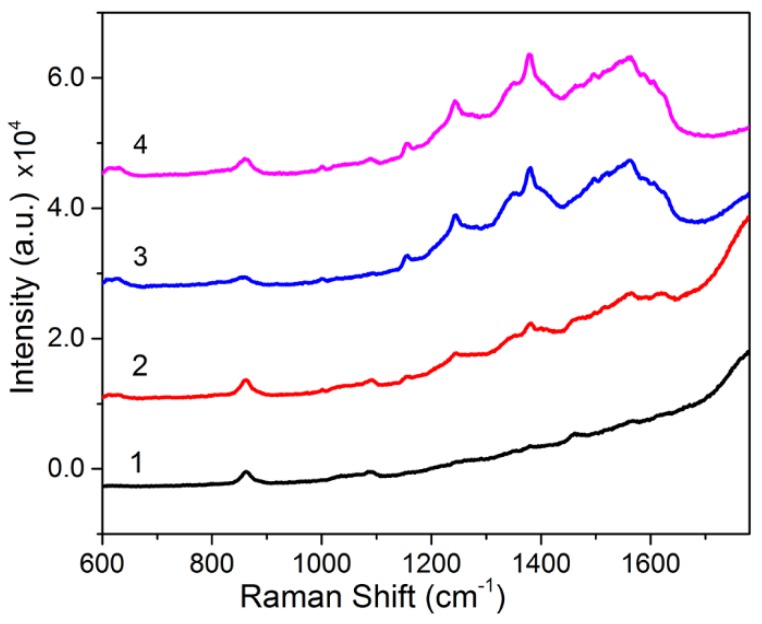
Surface-enhanced Raman scattering (SERS) spectra of melanin–Ag nanocomposites prepared with different volumes of silver–ammonia complex under 514.5 nm excitation: (1) 100 μL; (2) 200 μL; (3) 300 μL; (4) 400 μL.

Figure 2 shows the absorption spectra and Raman spectra of the nanomaterial within the UV-Vis range. The absorption spectra within the UV-Vis range shows a clear absorption peak of melanin–Ag near 460 nm, thereby indicating the formation of Ag nanostructures. The absorption value shows a slowing trend as the wavelength increases, and this trend is consistent with the changes in melanin. Raman characterization indicates that no Raman signal was detected for 20 μg/mL melanin at 514.5 nm laser irradiation, but very strong fluorescence was observed. After silver nanoparticles were attached, very clear SERS signals were detected, and the SERS spectral peaks belong to the molecular structure of melanin; e.g., 1256 cm^−1^ (δ(C–H), ν(C–O)), 1356 and 1380 cm^−1^ (C–C A_1g_ mode), 1500 cm^−1^ (ν(C=N)), 1562 cm^−1^ (ν(C=C)), 1590 cm^−1^ (C–C E_2g_ mode) [6,26]. This finding suggests that the strong SERS signals of melanin–Ag can be effectively used for the rapid detection and imaging of intracellular melanin. Moreover, such SERS signals are adjustable. By properly controlling the dose of silver ions in the reaction, melanin–Ag nanocomposites exhibiting different Raman enhancement features were obtained. As shown in Figure 3, the optimal SERS effect can be acquired at the silver–ammonia complex volume of 300 μL, which exhibits about 8.4-fold greater enhancement than that at 100 μL.

### 3.2. SERS Detection and Imaging of Intracellular Melanin

**Figure 4:**
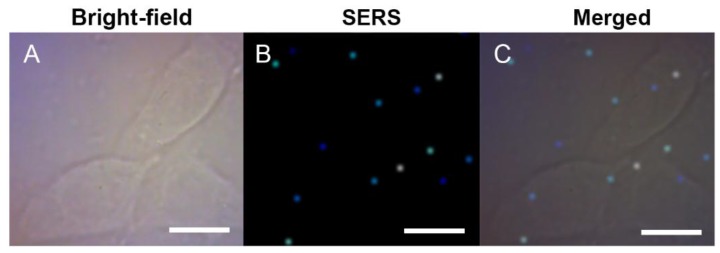

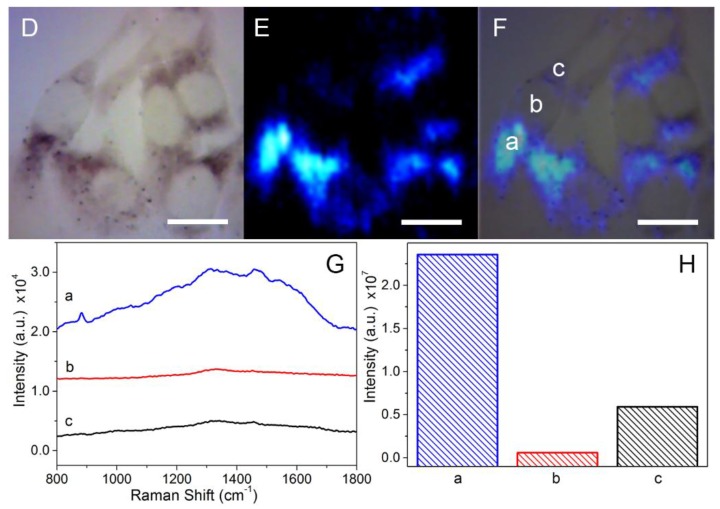
Raman imaging of B16-F10 cells (**A**–**C**) without or (**D**–**F**) with intercellular Melanin–Ag nanocomposites. (**A**,**D**) Typical bright-field images; (**B**,**E**) Raman images; and (**C**,**F**) the overlap images of B16-F10 cells. The SERS spectra of the different spots (a, b, and c) marked in (**F**) and corresponding quantitative data are shown in (**G**,**H**), respectively. Scale bar = 20 μm.

Test-tube experiments confirmed the reducibility of melanin for the synthesis of noble metal nanoparticles, and the produced melanin–Ag nanocomposites displayed good intrinsic SERS signals. In view of this, the nanocomposites were fabricated in situ in melanocytes, and the distribution and content of intracellular melanin was then detected by the SERS technique. Figure 4 shows the distribution of melaninin B16-F10 cells under SERS bioimaging. Under the Raman experimental conditions, conventional Raman imaging cannot obtain identifiable melanin Raman signals (Figure 4B). After melanin–Ag nanocomposites were built in situ in cells, the best imaging effect was achieved under the same experimental parameters for Raman scanning and for the 1000–1700 cm^−1^ range of SERS signals of melanin. SERS images matched well with the bright field images (Figure 4F). In addition, the imaging speed is fast, taking only 0.08 s per pixel; that is, only about 0.5 min is needed for a single cell. This speed far outpaces conventional Raman imaging techniques (tens of minutes and even several hours) [27,28]. The distribution of melanin SERS signals reveals that the signals of melanin mainly exist in the cytoplasm, while few are observed in the nucleus. This finding is consistent with the actual distribution of melanin in the cells. Figure 4G shows the SERS spectral lines sampled from different sites of B16-F10 cells. Within the region (a) from the cytoplasm to the nucleus (which is rich in endoplasmic reticulum), the acquired SERS spectral signal is very strong. However, only background signal is observed in the nucleus region (b). In the cytoplasm (c) at the distant end of the cell from the endoplasmic reticulum, a small amount of melanin is detected. These observations indicate that melanin is unevenly distributed in the cell. Quantitative analysis reveals that the integrated SERS intensity from 1000 to 1700 cm^−1^ of the spectral line-a is approximately four times that at site c (Figure 4H), further proving the inhomogeneous distribution of intracellular melanin.

### 3.3. SERS Monitoring of Intracellular Melanogenesis

**Figure 5:**
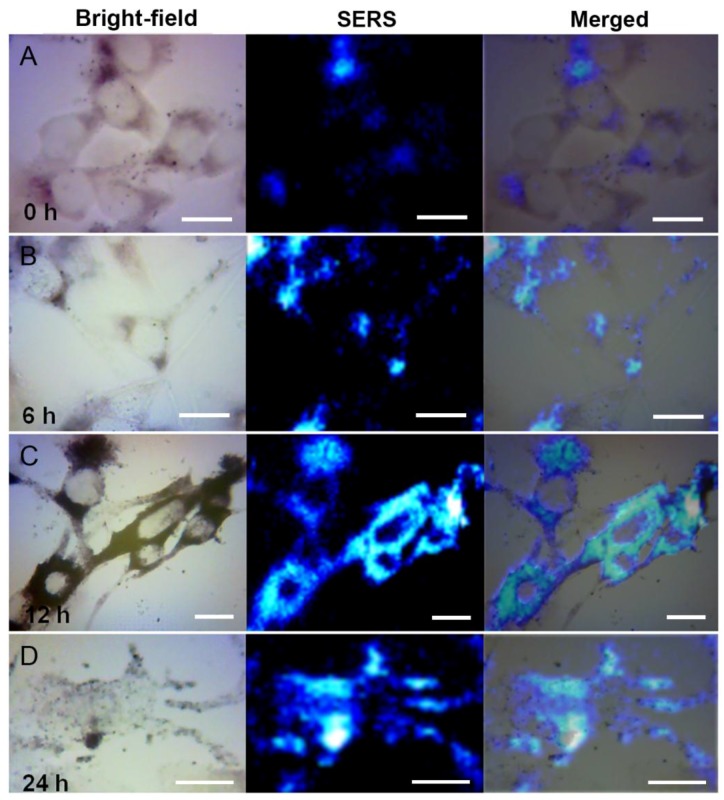
Surface-enhanced Raman scattering (SERS) monitoring of melanogenes is in 3-isobutyl-1-methylxanthine (IBMX)-treated B16-F10 cells. SERS images are acquired after IBMX treatment at (**A**) 0 h; (**B**) 6 h; (**C**)12 h; and (**D**) 24 h. Scale bar = 20 μm.

Exploring the generation of melanin in the cell is of great biological significance. IBMX is a common melanin-stimulating agent, and was used here to treat B16-F10 cells. At different periods of time, SERS technique was employed to analyze the generation of melanin. As shown in Figure 5, the morphologies of B16-F10cells without treatment with IBMX (0 h) were mainly elliptical and spindle-shaped, which is consistent with that of an immature melanocyte [29]. During this period, the synthetic amount of intracellular melanin is small and mainly distributes around the nucleus—specifically within the region of the rough endoplasmic reticulum where melanin is synthesized [30]. At 6 h after IBMX treatment, cell morphology starts to change significantly. Elongated dendrites start to form, thereby indicating that B16-F10 cells begin to mature under the action of the melanin-stimulating hormone. During this period, intracellular melanin is mainly distributed around the nucleus. However, the amount of melanin does not increase, and a small fraction starts to transfer out along the dendrites, thereby indicating that melanocytes mainly change in terms of morphology within the first 6 h. After that, the B16-F10 cells treated with IBMX experience a period of rapid biochemical changes, including the melanin content and the cellular contour. At 12 h, the cells form numerous thin dendrites. Simultaneously, a large amount of intracellular melanin is synthesized, and the SERS signal intensity of melanin in the endoplasmic reticulum is significantly enhanced. A good deal of melanin is also observed in the dendrites, indicating that melanin continued to move outside along the dendrites. At 24 h after IBMX treatment, the morphology of B16-F10 cells is significantly influenced, exhibiting less clear contours and with dendrites partially disconnected from the cell body. Intracellular melanin is mainly concentrated in the cell periphery, awaiting transportation from the cells. Part of the melanin is secreted in the form of membrane-bound vesicles [31]. Melanin secreted from the cells is also found in the extracellular matrix.

**Figure 6:**
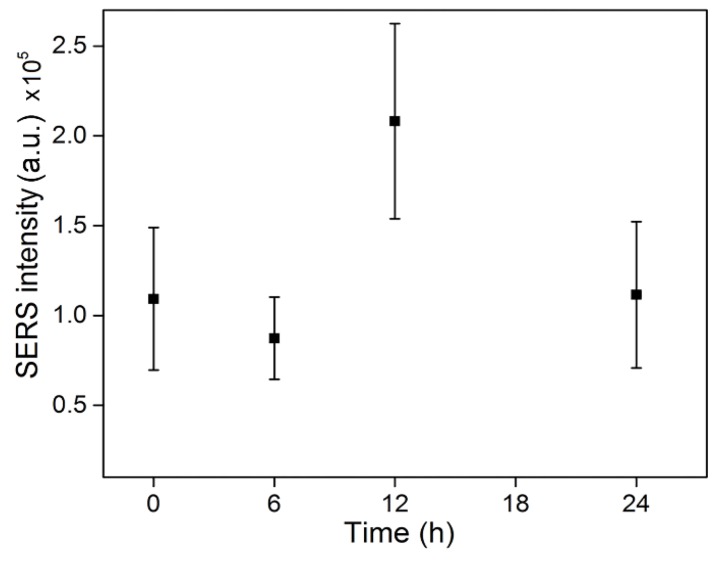
Mean SERS integrated intensities of the B16-F10 cells incubated with IBMX for 0, 6, 12, and 24 h. Data are expressed as the mean ± standard deviation.

Ten SERS spectral lines in the melanocytes were randomly extracted at different periods, and the average SERS integrated intensities were calculated to quantitatively analyze the changes of intracellular melanin. As shown in Figure 6, at 6 h after IBMX treatment, the SERS signal intensity of intracellular melanin weakened, probably because the cells during this period significantly changed in morphology, thereby producing numerous dendrites and enlarging the cell area. However, during this period, the synthetic amount of intracellular melanin is smaller, and a small amount of melanin move outside along the dendrites, thereby ultimately leading to a lower distribution density of melanin. At 12h after IBMX treatment, a large amount of melanin is synthesized, and the mean SERS density was 2.4 times greater than that at 6 h. After 24 h, the intracellular melanin significantly decreases, indicating that during this period the melanocytes mainly secreted melanin, but only a small amount of melanin was synthesized, despite having or synthesizing little melanin.

## 4. Conclusions

Melanin is an important molecule in the human body that performs essential biomedical functions. We firstly synthesized the melanin–Ag nanocomposites in the solution experiment without adding any other reducing agents. Moreover, as melanin is bonded with Ag nanoparticles, the Raman signals of melanin-Ag nanoparticles were also significantly enhanced. By using this method, the melanin–Ag nanocomposites were fabricated in situ intracellularly, and intracellular melanin was detected by Raman imaging based on the SERS features. This method featured high sensitivity and fast speed, and its scanning speed was much faster than that of conventional Raman imaging. Moreover, the content of intracellular melanin could be quantitatively analyzed by using SERS signals. The generation and secretion of intracellular melanin under the action of the melanin-stimulating hormone could also be successfully traced using this SERS technique. The proposed SERS technique in melanin detection can be a potentially powerful detection tool for detecting the biological functions of melanin and associated diseases.
